# CD62L expression marks SARS-CoV-2 memory B cell subset with preference for neutralizing epitopes

**DOI:** 10.1126/sciadv.adf0661

**Published:** 2023-06-14

**Authors:** Taishi Onodera, Nicolas Sax, Takashi Sato, Yu Adachi, Ryutaro Kotaki, Takeshi Inoue, Ryo Shinnakasu, Takayuki Nakagawa, Shuetsu Fukushi, Tommy Terooatea, Mai Yoshikawa, Keisuke Tonouchi, Takaki Nagakura, Saya Moriyama, Takayuki Matsumura, Masanori Isogawa, Kazutaka Terahara, Tomohiro Takano, Lin Sun, Ayae Nishiyama, Shinnya Omoto, Masaharu Shinkai, Tomohiro Kurosaki, Kazuo Yamashita, Yoshimasa Takahashi

**Affiliations:** ^1^ Research Center for Drug and Vaccine Development, National Institute of Infectious Diseases, Tokyo, Japan.; ^2^ KOTAI Biotechnologies Inc., Osaka, Japan.; ^3^ Tokyo Shinagawa Hospital, Tokyo, Japan.; ^4^ Laboratory of Lymphocyte Differentiation, WPI Immunology Frontier Research Center, Osaka University, Osaka, Japan.; ^5^ Shionogi & Co. Ltd., Osaka, Japan.; ^6^ Department of Virology I, National Institute of Infectious Diseases, Tokyo, Japan.; ^7^ Department of Life Science and Medical Bioscience, Waseda University, Tokyo, Japan.; ^8^ Laboratory of Viral Infection, Ōmura Satoshi Memorial Institute Graduate School of Infection Control Sciences, Kitasato University, Tokyo, Japan.; ^9^ Center for Infectious Diseases Education and Research, Osaka University, Osaka, Japan.

## Abstract

Severe acute respiratory syndrome coronavirus 2–neutralizing antibodies primarily target the spike receptor binding domain (RBD). However, B cell antigen receptors (BCRs) on RBD-binding memory B (B_mem_) cells have variation in the neutralizing activities. Here, by combining single B_mem_ cell profiling with antibody functional assessment, we dissected the phenotype of B_mem_ cell harboring the potently neutralizing antibodies in coronavirus disease 2019 (COVID-19)–convalescent individuals. The neutralizing subset was marked by an elevated CD62L expression and characterized by distinct epitope preference and usage of convergent V_H_ (variable region of immunoglobulin heavy chain) genes, accounting for the neutralizing activities. Concordantly, the correlation was observed between neutralizing antibody titers in blood and CD62L^+^ subset, despite the equivalent RBD binding of CD62L^+^ and CD62L^−^ subset. Furthermore, the kinetics of CD62L^+^ subset differed between the patients who recovered from different COVID-19 severities. Our B_mem_ cell profiling reveals the unique phenotype of B_mem_ cell subset that harbors potently neutralizing BCRs, advancing our understanding of humoral protection.

## Teaser

Phenotypic profiling of SARS-CoV-2 memory B-cells reveals neutralizing subset.

## INTRODUCTION

Severe acute respiratory syndrome coronavirus 2 (SARS-CoV-2) infection elicits humoral memory responses that confer protection via preexisting antibodies from long-lived plasma cells and recalled antibodies from restimulated memory B (B_mem_) cells (*1*). Secreted antibodies facilitate protection via multiple modes of action; for example, virus-neutralizing activities play important roles. The neutralizing antibody titers that exist before the infection are established as an immune correlate of protection over a few months after vaccination (*2*, *3*), but several lines of evidence suggest important contributions from recall responses in protective immunity, especially in modulating disease severity and resolving infection (*4*).

The major epitopes for neutralizing antibodies reside in the receptor binding domain (RBD) of the spike protein (*5*, *6*). RBD epitopes overlapping with angiotensin-converting enzyme 2 (ACE2)–binding sites [receptor binding site (RBS)] are the dominant targets for potent neutralizing antibodies (*7*, *8*). A single E484K mutation in representative RBS causes the profound reduction in RBD binding of polyclonal antibodies in coronavirus disease 2019 (COVID-19)–convalescent plasma (*9*, *10*), showing the immunodominance of the neutralizing epitopes that use RBS as antibody-contacting residues. The immunogenic nature of RBS-containing epitopes is probably due to the localization in the most distal parts of spike protein with less constrain on antibody recognition (*11*).

B_mem_ cells that preserve neutralizing antibodies are enriched by the RBD-binding activity of B cell antigen receptors (BCRs) on their surface, but not all of the cloned antibodies show detectable levels of neutralizing activity (*12*, *13*). Moreover, there is considerable divergence in the neutralizing potency (*14*). Given the heterogeneity of B_mem_ cell subsets with distinct phenotypes and functions (*15*–*19*), it is important to identify the B_mem_ phenotypic markers that enable us to enrich the cells with potently neutralizing antibodies (potent neutralizers) among RBD-binding B_mem_ cells. A large-scale phenotypic analysis of human B_mem_ cells has recently dissected the phenotypic features of B_mem_ cell subsets that have not been previously documented by conventional approaches (*18*–*20*). However, the links between phenotypic features and the functional outcomes of their BCRs remain poorly understood.

In this study, single-cell transcriptomic analysis was combined with monoclonal analysis to characterize the neutralizing potency of B_mem_ cell–derived antibodies. Analysis of COVID-19–convalescent individuals revealed surface CD62L expression as one of phenotypic characteristics of potent neutralizers. Comparative characterization of the antibody epitopes, functions, and immunoglobulin variable region gene repertoires between CD62L^+^ and CD62L^−^ B_mem_ cells provided the basis for the neutralizing activity. The CD62L^+^ subset, but not the CD62L^−^ subset from the same RBD-binding B_mem_ cells, correlated with neutralizing antibody titers in plasma and persisted in line with the disease severity. Thus, our functional marking of B_mem_ cells revealed a cell surface marker associated with neutralizing activity and disease severity.

## RESULTS

### Study design for linking transcriptional B_mem_ cell profiling with antibody function

SARS-CoV-2 B_mem_ cells were characterized using multiple parameters in a prospective cohort of COVID-19–convalescent individuals who had voluntarily agreed to a blood donation for B_mem_ cell profiling. All participants with symptomatic COVID-19 were initially confirmed by polymerase chain reaction (PCR) and seroconversion using anti-nucleocapsid antibodies. Symptoms were classified into mild, moderate, and severe categories based on their severity as previously reported (*10*). The participant demographics are summarized in fig. S1A. The period of symptom onset was between January and November 2020, before the initial detection of SARS-CoV-2 variants of concern in Japan (December 2020). Initial analysis was performed without stratification based on disease severity and days since disease onset owing to the limited numbers of donors.

**Fig. 1.**
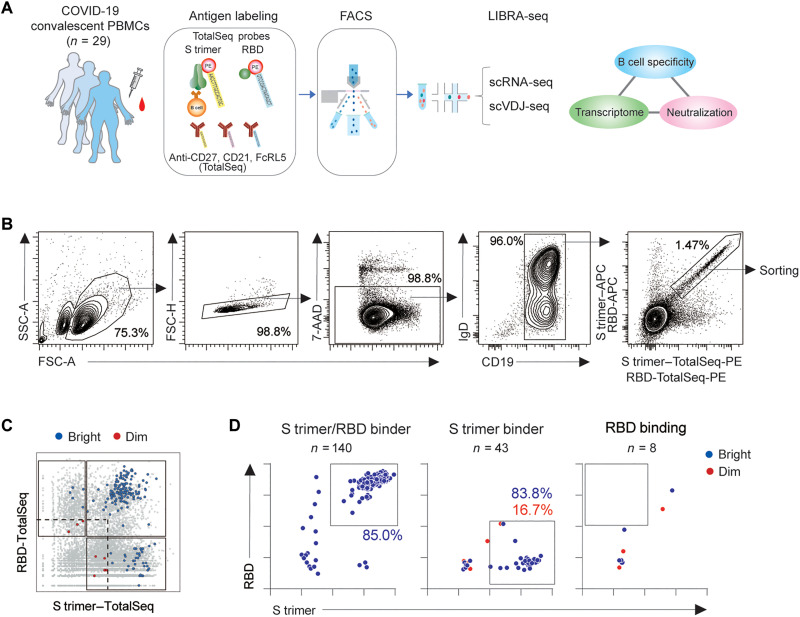
Linking transcriptional B cell profiling with antibody function. (**A**) Schematic diagram of experimental workflow for linking transcriptional B cell profiling with antibody function. COVID-19 convalescent peripheral blood mononuclear cells (PBMCs) were subjected to fluorescence-activated cell sorting (FACS) using DNA-barcoded antigen probes and single-cell transcriptome analysis (LIBRA-seq). The antibody neutralizing activities were determined using reconstructed mAbs and were linked to LIBRA-seq data. scRNA-seq, single-cell RNA sequencing; scV(D)J-seq, single-cell V(D)J sequencing. (**B**) FACS gating strategy for sorting before LIBRA-seq analysis. FSC, forward scatter; SSC, side scatter. (**C**) The barcode counts from antigen probes were plotted in a similar format to the FACS profile. Probe-positive cells were as gated, and bright and dim cells were defined as above and below 33 percentiles in the positive compartment (dashed line). Recombinant mAbs were reconstructed from bright and dim cells among S trimer^+^RBD^+^, non-RBD S trimer^+^, and RBD^+^ only (*n* = 191). (**D**) Antigen-binding activities of reconstructed mAbs were assessed by FACS using mAb-coated beads.

B cells from 22 donors, including 7 donors for two time points, were simultaneously labeled with stabilized S trimer and RBD probes coupled to a fluorescent dye for high-dimensional flow cytometry and DNA barcodes for single-cell transcriptome analyses coupled with BCR specificity [linking BCR to antigen specificity through sequencing (LIBRA-seq)] (Fig. 1A) (*21*–*23*). The cells from healthy donors (*n* = 5) were also included for reference. Although this labeling approach may cause potential bias toward high-affinity B cells, it does not critically affect the outcome of this study, which is intended to compare the phenotypic characteristics between functional (neutralizing) and nonfunctional cells among RBD-binding cells. Flow cytometric profiling of convalescent and prepandemic samples revealed increased numbers of probe-positive cells in convalescent samples (fig. S1B), thereby validating probe specificity. After magnetic enrichment and flow cytometric sorting of S trimer–binding cells (Fig. 1B), cells were subjected to LIBRA-seq analysis.

Recombinant immunoglobulin G1 (IgG1) monoclonal antibodies (mAbs) were created on the basis of IgV gene sequences from the transcriptome data, and their neutralizing activities were quantified using the vesicular stomatitis virus (VSV)–pseudo-typed virus assay. By combining transcriptome data with neutralization activity from the same cell, we linked antibody function with the BCR repertoire/gene expression profiling at the single-cell level. We collected single-cell transcriptome data from 9474 cells from 29 specimens (including specimens from seven donors on their second visit). Four populations were visualized on the basis of the signal intensities from the S trimer and RBD probes (Fig. 1C). In total, 191 B cell clones from three populations (S trimer binding, RBD binding, and S trimer/RBD binding) were selected to create mAbs to validate antibody reactivity against S trimer and RBD proteins. The antibodies reconstructed from the S trimer/non-RBD–binding and S trimer/RBD–binding populations with bright intensity reproduced the original binding specificity for S trimer binding (83.8%) and S trimer/RBD double binding (85%) (Fig. 1D). However, the reconstructed IgG1 specificity from the dim cells for S trimer binding was only 16.7%. Furthermore, reconstructed IgG1 (*n* = 8) from cells binding only RBD did not reproduce RBD specificity. Therefore, we focused on S trimer^+^RBD^+^ cells (RBD binder) and non-RBD S trimer^+^ cells (non-RBD binder) with a bright intensity.

### Multiple B cell clusters with distinct transcriptional profiling

**Fig. 2.**
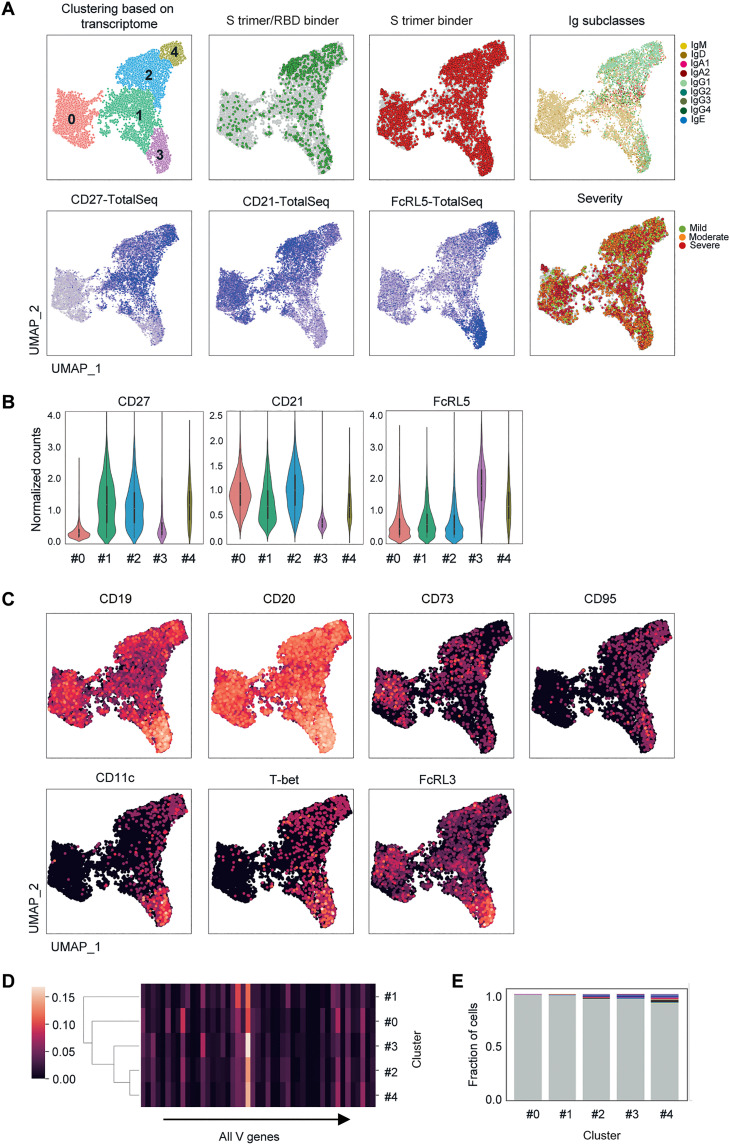
Multiple B cell clusters with distinct transcriptional profiling. (**A**) UMAP plot displaying the cells defined by their single-cell transcriptome analysis. The cells are colored based on their cluster assignments by the Louvain clustering algorithm. The distribution of S trimer^+^RBD^+^, non-RBD S trimer^+^, and immunoglobulin subclasses was highlighted (top). The distribution of CD27^+^/CD21^+^/FcRL5^+^ cells and cells with different disease severities was highlighted in different colors (bottom). (**B**) The normalized counts for the indicated transcripts are shown in the violin plot from each cluster. (**C**) The distribution of the indicated transcripts for each cluster is highlighted in different colors. (**D**) Heatmap representation of V_H_ gene fraction for each cluster. (**E**) Cells in public BCR clonotypes for each cluster are highlighted in colors. Different colors indicate different donors.

Transcriptional profiling using LIBRA-seq analysis revealed five distinct cell clusters using a uniform manifold approximation and projection (UMAP) plot (Fig. 2A). Whereas the S trimer binders were dispersed into five clusters (Fig. 2A), the RBD binders were located more intensively in clusters 2 to 4 than in other clusters (Fig. 2A). IgG1-switched B_mem_ cells also colocalized in clusters 2 to 4, suggesting their preferential localization. Cluster 0 comprised numerous IgM^+^/CD27^−^/CD21^+^ cells on DNA-barcoded anti-CD27 and CD21 antibody staining (Fig. 2, A and B), indicating that this cluster mainly contained naive B cells. Likewise, the cells in cluster 1 were IgM^+^/CD27^+^/CD21^+^, indicative of IgM^+^ B_mem_ cells. In contrast, clusters 2 to 4 mostly harbored IgG1-switched B_mem_ cells that were surface CD27^+^/CD21^+^, CD27^−^/CD21^−^/FcRL5^+^, or CD27^+^/CD21^low^/FcRL5^low^, respectively. Given the surface expression profiles of CD27/CD21/FcRL5, cluster 3 likely contains atypical B_mem_ cells (CD27^−^/CD21^−^/FcRL5^+^), cluster 2 cells are resting or classical B_mem_ cells (CD27^+^CD21^+^), and cluster 4 cells phenotypically resemble activated B_mem_ cells (CD27^+^CD21^low^) (*16*). CD27^+^CD21^low^ B_mem_ cells may also include recent germinal center (GC) descendants primed for plasma cell differentiation or newly differentiated B_mem_ cells (*24*–*26*). The cluster distribution was not noticeably different between the mild, moderate, and severe groups, albeit with a limited number of donors (Fig. 2A).

In-depth phenotypic analysis using high-dimensional mass or flow cytometry has been performed on total human B cells without selecting BCR specificity nor isotypes (*18*–*20*). Multiple B_mem_ cell subsets identified in the previous studies may be the same or partially overlapping with the clusters in this study. Atypical or effector-like B_mem_ cell subsets can be marked as CD95^+^ or CD19^high^CD11c^+^ B_mem_ cells (*20*). The expression of sub-phenotyping markers [CD19 (*CD19*), CD20 (*MS4A1*), CD73 (*NT5E*), CD95 (*FAS*), CD11c (*ITGAX*), T-bet (*TBX21*), and FcRL3 (*FCRL3*)] could be assessed by our transcriptome readout. Therefore, the transcriptional profiles of these additional markers were plotted by UMAP (Fig. 2C).

*CD19*, *MS4A1*, and *ITGAX* transcripts were highly expressed in cluster 3, supporting the overlap of cluster 3 with CD19^high^CD11c^+^ B_mem_ cells. The CD19^high^CD11c^+^ B_mem_ cells also shared the features of T-bet^+^ B cells, and more abundant *T-bet* expression was confirmed in the cluster 3. *FAS* expression may define effector B_mem_ cell subset (CD95^+^ B_mem_ cells) that is highly responsive to BCR and CD40 signaling in vitro (*20*); however, *FAS* transcript in our UMAP plot was widely detected across clusters 2 to 4, with higher density in clusters 3 and 4. The CD95^+^ B_mem_ cell subset is heterogeneous in the expression of CD73 and IgM/G/A isotypes (*20*). Along with the *IgG* transcript data, the paucity of *NT5E* transcripts in cluster 4 probably marks this cluster as the CD73^−^ subpopulation of the CD95^+^ B_mem_ cell subset. IgG^+^CD73^+^ subpopulation of the CD95^+^ B_mem_ cell subset may be included in the cluster 2 in addition to classical B_mem_ cells.

Intercluster comparisons of IgV gene usage failed to reveal any cluster-specific V genes in either non-RBD binders or RBD binders (fig. S2A). However, the phylogenetic relationship between BCR repertoires based on the overall similarity of V_H_/V_L_ (variable region of immunoglobulin heavy/light chain) gene sequences demonstrated the closest relationship was between clusters 2 and 4 (Fig. 2D). Antibody clonotypes shared among multiple donors were defined as public clonotypes and are highlighted in the UMAP plot (fig. S2B). The cells in cluster 4 used public clonotypes at the highest frequency (Fig. 2E). The cells in clusters 2 and 3 also used public clonotypes at higher frequencies than those in clusters 0 and 1. The enrichment of public clonotypes in clusters 2 to 4 is consistent with the dominant localization of switched B_mem_ cells in these clusters, as switched B_mem_ cells, mostly of the IgG1 subclass, express convergent BCR repertoires (*12*). Classical B_mem_ (cluster 2), activated B_mem_ (cluster 4), and IgM^+^ B_mem_ (cluster 1) cells carried a high number of somatic hypermutations compared to naive (cluster 0) and atypical B_mem_ (cluster 3) cells (fig. S2C).

### B cell mapping with potently neutralizing antibodies reveals CD62L phenotype

**Fig. 3.**
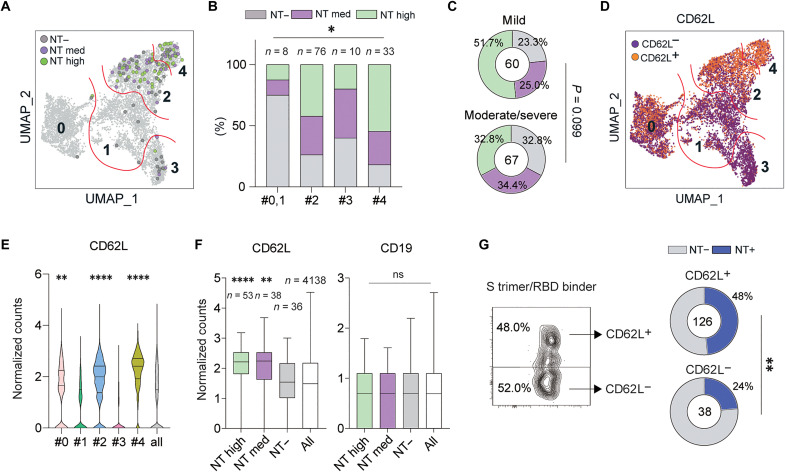
B cell mapping with potently neutralizing antibody identifies CD62L phenotype. (**A**) RBD binders with bright intensity (*n* = 127) were tested for the neutralizing activities and highlighted in UMAP based on the NT activities (NT high and NT med). (**B**) Percentages of the indicated NT activities are shown in cluster 0/1 (*n* = 8), 2 (*n* = 76), 3 (*n* = 10), and 4 (*n* = 33). (**C**) Distribution of NT activities in different disease severities. (**D**) Distribution of CD62L^+^ and CD62L^−^ cells is highlighted in UMAP. (**E**) Expression levels of CD62L among the cells in different clusters are shown on the basis of the transcript counts. Statistical difference was evaluated between the cells in each cluster and all cells. (**F**) CD62L and CD19 expression levels are comparatively plotted on the basis of neutralizing activities. Statistical difference was evaluated between the cells in each group and all cells. (**G**) S trimer^+^RBD^+^IgG^+^ B_mem_ cells were fractionated into either CD62L^+^ or CD62L^−^ subset and subjected to single-cell culture. Pie charts represent the ratios of mAb clones with NT activity. Statistical analyses were performed using the chi-square test in (B) and (C), Kruskal-Wallis test followed by Dunn’s multiple comparison test in (E) and (F), Fisher’s exact test in (G), and Jackknife resampling was performed to assure the statistical significance in (G). *P < 0.05, **P < 0.01, and ****P < 0.0001. ns, not significant (*P* ≥ 0.05).

Given the biased localization of class-switched RBD binders in clusters 2 to 4, we focused on these three clusters in the following analysis. Recombinant IgG1 antibodies from non-RBD binders and RBD binders with bright intensities (*n* = 127) were tested for neutralizing activity (Fig. 3A). Our selection criteria for creating the IgG1 panel were based on the S trimer binding intensity, broad coverage from multiple donors, and clonotypes; therefore, the numbers of evaluated IgG1 antibodies were not equally distributed among the clusters. Only 20 to 33% of the reference antibodies prepared from clusters 0 and 1 exhibited neutralizing activity, confirming the minor representation of neutralizing antibodies in these clusters. In contrast, more than half of mAbs from clusters 2 to 4 exhibited neutralizing activity [median inhibitory concentration (IC_50_) < 5 μg/ml], and 42 and 55% of mAbs from clusters 2 and 4, respectively, were identified as potent neutralizers (IC_50_ < 0.1 μg/ml, NT high) (Fig. 3B). The IgG1 panel was also subgrouped on the basis of disease severity in the donors (Fig. 3C). IgG1 from patients in the mild group (*n* = 60) tended to show more potent neutralizing activity than IgG1 from patients in the moderate/severe groups (*n* = 67), although the difference was not statistically significant.

To identify the phenotypic markers of potent neutralizers, we searched for genes that were more abundantly expressed in potent neutralizers among clusters 2 and 4. We focused on CD62L (*SELL*), which was broadly expressed in clusters 4, 2, and 0, but showed the highest levels in cluster 4 based on the transcriptome data (Fig. 3, D and E). Consistently, high surface expression of CD62L was observed in IgG^+^CD95^+^ and CD45RB^−^ B_mem_ cell subsets that appear to be partially overlapped with the clusters 2 and 4 in this study, but not in the CD19^high^CD11c^+^ B_mem_ cell subset nor conventional B_mem_ cell subsets (*19*, *20*). Notably, the CD62L^+^ B_mem_ cell subset is also induced among flu-binding B cell fraction by booster vaccination, with earlier kinetics than other B_mem_ cell subsets (*15*); however, the CD62L^+^ subset remains to be characterized in relation to the functionality of their BCRs. Therefore, we chose CD62L as a candidate marker for neutralizing B_mem_ cells. The expression levels of CD62L transcripts, as well as CD19 as the reference, were plotted for a potent neutralizer (IC_50_ < 0.1 μg/ml, NT high), a neutralizer (0.1 < IC_50_ < 5 μg/ml, NT med), and a non-neutralizer (IC_50_ > 5 μg/ml, NT−) (Fig. 3F). The NT+ group (NT high and NT med), but not the NT− group, showed higher expression levels of CD62L transcripts than all transcriptome antibodies (Fig. 3F), whereas such differential expression was not observed for CD19 transcripts.

To evaluate whether the neutralizers are enriched in surface CD62L^+^ cells over CD62L^−^ cells, we fractionated S trimer^+^RBD^+^IgG^+^ B_mem_ cells among the CD21^+^CD27^+^ phenotype into CD62L^+^ and CD62L^−^ populations using flow cytometry and then performed single-cell cultures (Fig. 3G and fig. S3A). The supernatants were then subjected to a high-throughput neutralization assay. Peripheral blood mononuclear cells (PBMCs) were collected from patients with mild disease, as shown in fig. S3B. Because this assay is affected by IgG concentrations in the culture supernatant, we initially compared the IgG concentrations from the different populations, and there was no bias on the IgG concentrations between CD62L^+^ and CD62L^−^ cell origin or between NT^+^ and NT^−^ (fig. S3, C and D). Under these conditions, the antibodies cloned from CD62L^+^ B_mem_ cells harbored the neutralizing antibodies at elevated frequency compared with CD62L^−^ B_mem_ cells (Fig. 3G). Thus, the RBD-binding IgG^+^ compartments among the CD21^+^CD27^+^ phenotype, a subset known to be a major source of neutralizing BCRs, can be further subgrouped into the neutralizers based on surface CD62L expression.

### V_H_ gene usage, mutational content, and epitope focusing in the CD62L^+^ B_mem_ cells

**Fig. 4.**
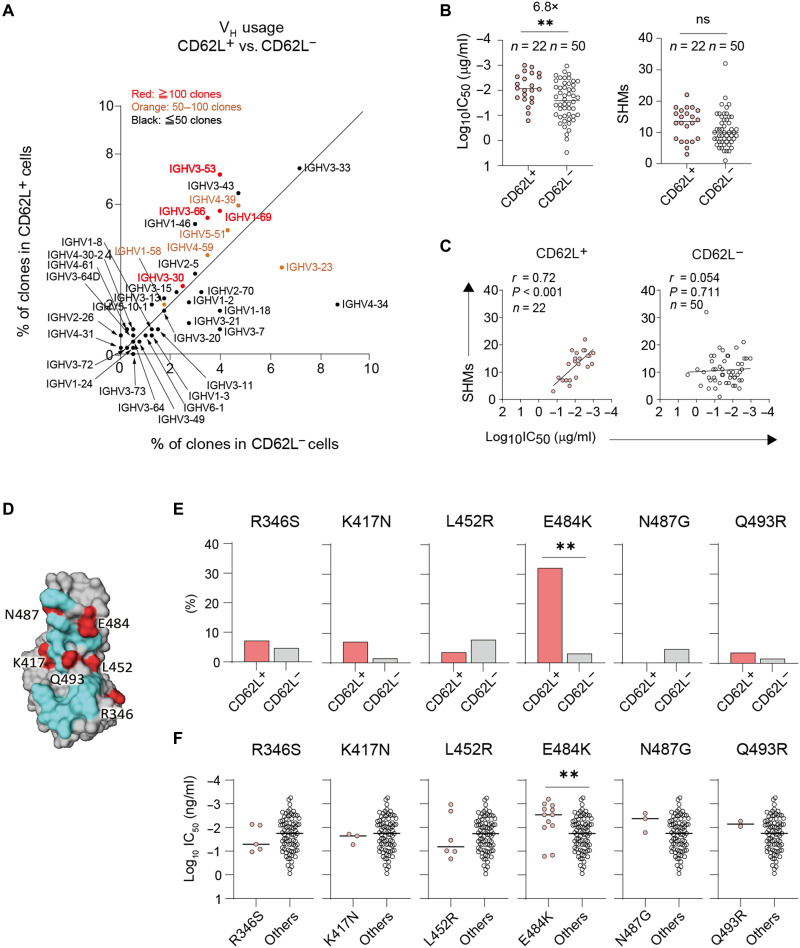
IgV genes and epitope preference of CD62L^+^ B_mem_ subset. (**A**) Numbers of V_H_ genes detected from the single-cell transcriptome analysis were plotted in IgG1^+^CD62L^+^ and CD62L^−^ B_mem_ subsets among RBD binders (*n* = 782). The V_H_ genes were highlighted on the basis of the number of sequences deposited into the CoV-AbDab database (https://opig.stats.ox.ac.uk/webapps/covabdab/). (**B**) IC_50_ and numbers of somatic hypermutations (SHMs) per V_H_ + V_L_ genes from RBD-binding, NT+ IgG clones derived from IgG^+^CD62L^+^ (*n* = 22) and CD62L^−^ (*n* = 50) B_mem_ cell subsets were plotted. Each dot represents the data from an individual IgG clone. (**C**) Spearman correlations between IC_50_ and somatic hypermutations in each subset are plotted. *R* and *P* values are indicated. (**D**) RBD structure (Protein Data Bank: 6LZG) highlighted with human ACE2 binding region (light blue) and amino acid positions of single-mutant RBDs (red) was shown. (**E**) IgG clones derived from the indicated B_mem_ cells were subjected to the binding assay against six RBD mutants bearing a single mutation. The IgG clones that lost the binding to the indicated RBD mutants only were identified and the percentages are shown. (**F**) NT activities of IgG clones that lost the binding to RBD mutants were comparably plotted with other IgG clones. Statistical analyses were performed using Spearman’s rank-order correlation test in (B), Fisher’s exact test in (D), and Mann-Whitney test in (B) and (E). **P < 0.01.

To gain mechanistic insights into the neutralizing activity of the CD62L^+^ B_mem_ cell subset, we compared V_H_ gene usage of RBD binders (*n* = 782) between *SELL^high^* and *SELL^low^* groups. V_H_ gene segments were plotted on the basis of the number of clones detected from two groups (Fig. 4A). After searching the CoV-AbDab database, the convergent V_H_ genes isolated from multiple donors as neutralizing clones were highlighted if they were deposited above the indicated frequencies. Multiple convergent V_H_ genes were used in the *SELL^high^* group more frequently than the *SELL^low^* group. Thus, the frequent use of convergent V_H_ genes genetically supports the enrichment of potent neutralizers in the CD62L^+^ population.

Next, recombinant mIgGs were created for the detailed characterization of the neutralizing activities and epitopes. To this end, we were able to generate CD62L^+^-derived (*n* = 22) and CD62L^−^-derived mAbs (*n* = 50) cells. The neutralizing activities (IC_50_) and somatic hypermutations of the monoclonal IgG panel were quantitated. IC_50_ of CD62L^+^-derived antibodies were lower than those of CD62L^−^-derived ones (Fig. 4B), confirming that CD62L^+^ B_mem_ cells are enriched with potent neutralizers. Despite such higher neutralizing activities, the numbers of mutations between CD62L^+^ and CD62L^−^ cells were comparable (Fig. 4B). The neutralizing activities and somatic hypermutations of RBD-binding antibodies do not correlate well each other, probably reflecting the heterogenicity of the RBD epitopes (*8*, *14*, *27*). Here, by dividing RBD-binding antibodies based on their CD62L^+/−^ origins, we found a clear correlation between neutralizing activity and somatic hypermutations in CD62L^+^ cells but not CD62L^−^ cells (Fig. 4C). These data suggest that CD62L^+^ cells recognize more homogeneous epitopes than CD62L^−^ cells, thereby increasing the mutation dependency of their neutralizing activities.

To further gain insights into the epitopes for CD62L^+^ B_mem_ cells, we prepared six RBD mutants that had a single mutation in the neutralizing epitopes, as used in a previous study (Fig. 4D) (*28*), and the binding profiles of the monoclonal IgG panel to the RBD mutants were examined. Notably, the frequencies of IgG clones that lost binding to the E484K mutation, but not to the other mutations, were considerably different between the CD62L^+^ and CD62L^−^ subsets (Fig. 4E). E484 localizes to the immunodominant site of RBDs and often constitutes a key footprint of potently neutralizing antibodies (*8*, *29*). The higher potency of the IgG clones that lost binding via the E484K mutation was supported by the 10-fold lower IC_50_ compared with other NT^+^ IgG clones (Fig. 4F). These results suggest that the high potency of CD62L^+^ IgG clones is, at least in part, ascribed to the preferential targeting of neutralizing epitopes that include E484 as a key footprint. On the other hand, the resistance to E484K and other mutations in CD62L^−^ IgG clones suggest more flexible RBD recognition that is not solely governed by E484 or the listed single mutation. Together, CD62L expression marks B_mem_ cells that preferentially recognize immunodominant and neutralizing epitopes via BCRs of convergent V_H_ gene usage.

### Relationship between the CD62L^+^ B_mem_ cell subset and plasma neutralizing antibody concentration/disease severity

**Fig. 5.**
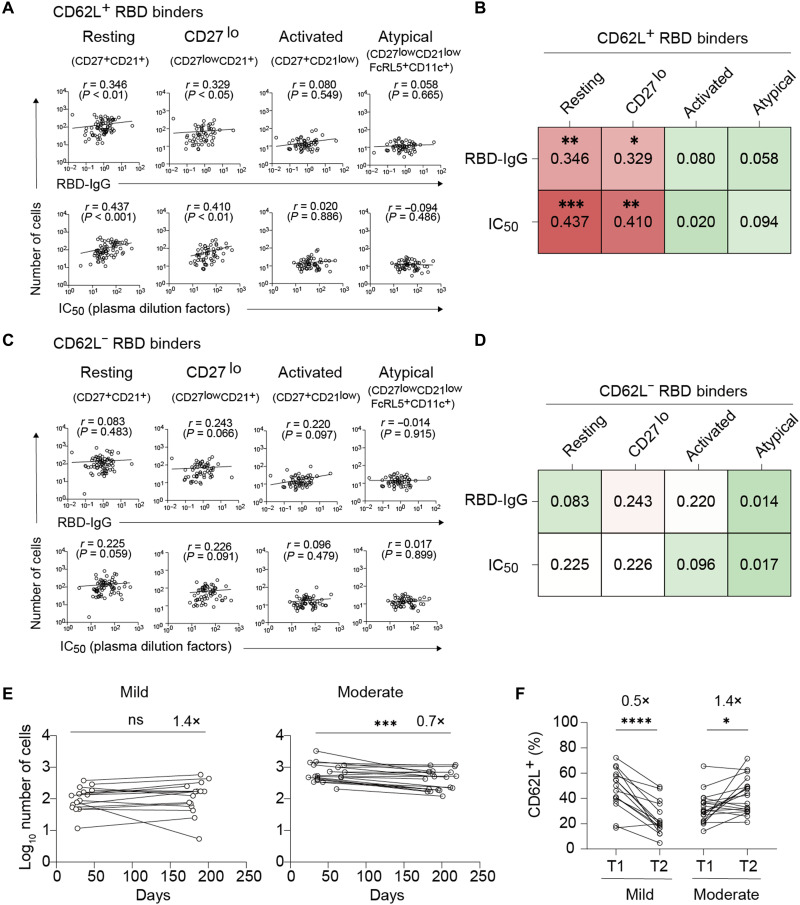
The functional relevance of CD62L^+^ B_mem_ cell subset. (**A** to **D**) RBD-binding IgG^+^ B_mem_ cells were divided into four populations (resting, CD27^lo^, activated, and atypical). The gating strategies for the B_mem_ cell subsets are shown in fig. S4. The numbers of CD62L^+^ (A and B) or CD62L^−^ (C and D) subsets in individual populations were plotted against RBD-binding IgG tiers or plasma dilution factors for IC_50_. (**E**) The numbers of RBD-binding IgG^+^ B_mem_ cells were enumerated in the T1 and T2 periods in the mild and moderate patients. (**F**) CD62L^+^ ratios among the RBD-binding IgG^+^ B_mem_ cells were plotted in the T1 and T2 from mild and moderate patients. Each dot represents the data from an individual donor. Statistical analyses were performed using the Spearman’s rank-order correlation test in (A) to (D), Wilcoxon test in (E) and (F). *P < 0.05, **P < 0.01, ***P < 0.001, and ****P < 0.0001.

To assess the possible link between the CD62L^+^ B_mem_ cell subset and circulating antibodies, we quantified the B_mem_ cell subsets and antibody titers side by side with a larger cohort (*n* = 74, 6 months after the disease onset) (Fig. 5, A to D). The prior infection in this large cohort was confirmed by anti-nucleocapsid seropositivity. RBD-binding IgG^+^ B_mem_ cells were subdivided into CD27^+^CD21^+^ (resting), CD27^low^/CD21^+^ (CD27^low^), CD27^+^CD21^low^ (activated), and CD27^low^CD21^low^CD11c^+^FcRL5^+^ (atypical) with or without CD62L expression (fig. S4) (*16*, *17*, *30*). No correlation was found between the antibody titers and the activated or atypical B_mem_ cell subset; however, we did observe a correlation in the CD62L^+^ subset among the resting and CD27^low^ cells (phenotypically similar to those cells in clusters 2 and 4) with neutralizing antibodies rather than RBD-binding antibodies (Fig. 5, A and B). Notably, this correlation was lost in the CD62L^−^ subset (Fig. 5, C and D). The more profound correlation between the CD62L^+^ B_mem_ cell subset and neutralizing antibodies over RBD-binding antibodies further supports the skewing of B_mem_ cells toward neutralizing epitopes in cells expressing the surface CD62L marker.

Last, to assess the possible relevance of the CD62L^+^ B_mem_ cell subset on disease severity, we longitudinally tracked the development and persistence of the CD62L^+^ and CD62L^−^ subsets in the RBD-binding IgG^+^CD21^+^CD27^+^ memory compartment in COVID-19–convalescent individuals who had recovered from mild or moderate diseases (mild, 16 donors; moderate, 20 donors; fig. S5). The number of RBD-binding IgG^+^ B_mem_ cells remained unchanged up to the T2 period (Fig. 5E), but the number of B_mem_ cells slightly decreased (0.7-fold) in moderate patients. Despite the quantitative stability of IgG^+^ B_mem_ cells during the observed time period, the ratio of CD62L^+^ cells markedly changed with time, dependent on disease severity (Fig. 5F). The CD62L^+^ ratios slightly increased in the moderate group from T1 to T2; however, the CD62L^+^ subset in the mild group decreased by twofold at T2. Thus, the disease severity affected the kinetics of CD62L^+^ B_mem_ cells.

## DISCUSSION

SARS-CoV-2–neutralizing antibodies in circulation and those expressed on B_mem_ cells have >1000-folds variation in the neutralizing potency, even after the RBD-binding antibodies are selected. A wide range of antibody-binding affinities contributes to the variation, but it is further exaggerated by the heterogeneity in the RBD epitopes that are positioned in divergent sites of RBDs. Hence, the binding affinities and neutralizing activities of RBD antibodies often do not correlate even under monoclonal settings (*8*, *14*, *27*). Because B_mem_ cells are selected within the GC by BCR affinities to the antigens and not by neutralization activities, the links between the B_mem_ cell phenotypes and the functionality of BCRs have not been fully evaluated. Here, we provided the evidence that the potently neutralizing B_mem_ cell subset is enriched in the CD62L^+^ B_mem_ cell subset, which is repeatedly observed in human B_mem_ cells but lacking detailed characterization. The enrichment of neutralizers by the CD62L subsetting was about twofold; however, 6.8-fold increase of neutralizing activities (IC_50_) and selective correlation with neutralizing antibody titers in CD62L^+^ subset reinforces the significance of CD62L subsetting,

CD62L^+^ B_mem_ cell subset is found as one of phenotypically distinct subset among flu-binding B_mem_ cells and designated as activated memory 1 (AM1). This B_mem_ cell subset promptly emerges in the peripheral blood following influenza booster vaccination and declines more rapidly than other B_mem_ cell subsets (*15*). Such transient circulation of the AM1 subset may be analogous to less persistence of CD62L^+^ B_mem_ cell subset than CD62L^−^ counterpart within the COVID-19 mild patients in this study. We provided evidence that the CD62L^+^ subset emerged in patients with COVID-19 frequently carries BCRs against neutralizing epitopes that use E484 as key amino acid. E484 positioning in unmasked ACE2-binding face of RBD “left shoulder” presumably allows the antibody recognition with less structural constrains than other RBD sites (*11*). Mapping of RBD mutations that affect recognition by polyclonal plasma antibodies identifies E484 as an immunodominant site on RBD (*9*, *10*), with a single E484K mutation causing threefold reduction in the RBD binding among multiple convalescent donors. Therefore, the E484-focused RBD recognition suggests that the CD62L^+^ subset is derived from B cells that receive stimulation by more immunogenic epitopes after infection. The rapid decline from circulation implies their death or recruitment into the tissue where they contribute to neutralizing antibody production. The correlation between CD62L^+^ subset and circulating neutralizing antibody titer supports the latter possibility. We speculate that the CD62L^+^ phenotype probably marks the circulating B_mem_ cell subset that is primed by immunogenic epitopes and then recruited from the primed site to the effector tissue.

How neutralizing BCRs are preferentially preserved in the CD62L^+^ B_mem_ cell subset remains key question. The numbers of somatic hypermutations have been used for an indicative marker for how intensively the B_mem_ precursors are subjected to affinity maturation process within GC before the B_mem_ cell formation (*31*). Given the comparable mutational contents between CD62L^+^ and CD62L^−^ B_mem_ cells, the GC-dependent maturation process during the formation of two B_mem_ cell subsets appears to be similar. The binding epitopes, other than the binding affinity, are important determinants for the neutralizing activities. In this context, we consider that the focused binding to immunogenic E484 epitopes by CD62L^+^ B_mem_ cell subset is one of the possible determinants for more potent neutralizing activities. In this scenario, surface CD62L protein may be an activation marker, rather than a constitutive marker for distinct B cell lineage, that is indicative for the recent stimulation by immunogenic epitopes. CD62L expression in B cells is enhanced by forkhead box protein O1 (FOXO1) transcriptional factor, the key mediator of B cell selection (*32*–*34*). Thereby, the biased preservation of neutralizing BCRs in CD62L^+^ B_mem_ cell subset could reflect CD62L expression via FOXO1-mediated pathway in the cells that receive strong T helper signals after the stimulation by immunogenic epitopes.

In this study, we have enrolled the patients with COVID-19 who were likely infected by ancestral strains before the emergence of antibody-escape variants, and the homologous B_mem_ cell responses to Wuhan RBD were analyzed. E484-containing epitopes are immunogenic and recognized by potently neutralizing antibodies in this experimental setting; however, upon the emergence of many variants with E484K/A mutations, such as Omicron variants, the antigenic map of RBD epitopes targeted by neutralizing antibodies is significantly changed (*35*–*37*). It is possible that the CD62L^+^ B_mem_ cell subset shows reduced neutralizing activity to the emerged E484K/A^+^ variants. In addition, it is important to determine to what extents the findings in this study are applied to the BCR function of B_mem_ cells that are either primed or boosted by antigenically distinct variants through breakthrough infection or Omicron-adapted vaccination.

## MATERIALS AND METHODS

### Human samples

SARS-CoV-2–infected individuals (confirmed by SARS-CoV-2 PCR on nasopharyngeal swab samples upon admission) were enrolled at the Tokyo Shinagawa Hospital and Tokyo Center Clinic. Blood samples were collected from 118 convalescent individuals. None of the convalescent individuals were reinfected with SARS-CoV-2 or subjected to any COVID-19 vaccine during the course of sampling. Symptoms were grouped into mild (*n* = 89), moderate (*n* = 26), and severe (*n* = 9) based on severity [mild, no pneumonia; moderate, 93% < oxygen saturation (SpO_2_) < 96%; severe, SpO_2_ ≤ 93%]. Prepandemic blood samples from healthy individuals were collected before the COVID-19 pandemic (August 2018 to July 2019) at the Japanese Red Cross. Blood was collected in Vacutainer CPT tubes (BD Biosciences) and centrifuged at 1800*g* for 20 min. PBMCs were suspended in plasma and harvested into different tubes, followed by centrifugation at 300*g* for 15 min. After the plasma was transferred into another tube, the PBMC pellets were washed three times with phosphate-buffered saline (PBS) before cryopreservation in CELLBANKER 1 (Zenoaq). The plasma samples were further centrifuged at 800*g* for 15 min and transferred into another tube to completely remove PBMCs. The plasma samples were heat-inactivated at 56°C for 30 min before use.

### Cells

Expi293F cells were maintained in accordance with the manufacturer’s instructions (Thermo Fisher Scientific). VeroE6/TMPRSS2 cells (JCRB1819, JCRB Cell Bank) were maintained in low-glucose Dulbecco’s modified Eagle’s medium (DMEM) (Fujifilm) containing 10% heat-inactivated fetal bovine serum (FBS) (Biowest), geneticin (1 mg/ml; Thermo Fisher Scientific), and penicillin/streptomycin (100 U/ml; Thermo Fisher Scientific) at 37°C supplied with 5% CO_2_.

### Recombinant S antigens

The human codon-optimized nucleotide sequence coding for the S protein of the CoV2 isolate (GenBank: MN994467) was commercially synthesized (Eurofins Genomics). The RBD (amino acids 331 to 529), along with the signal peptide (amino acids 1 to 20; MIHSVFLLMFLLTPTESYVD) plus a histidine tag and an Avi-tag, was cloned into the mammalian expression vector pCAGGS. A soluble version of the S protein (amino acids 1 to 1213), including a T4 foldon trimerization motif, histidine tag, and Avi-tag, was cloned into the mammalian expression vector pCMV. The S protein sequence was modified to remove the polybasic cleavage site (RRAR to A), and two stabilizing mutations were introduced (K986P and V987P; wild-type numbering) (*38*). RBD mutants R346S, K417N, L452R, A475V, E484K, N487G, Q493R, and N501Y were generated by site-directed mutagenesis using primers with relevant nucleotide substitutions and Gibson assembly (New England Biolabs). Recombinant proteins were produced using Expi293F cells, according to the manufacturer’s instructions (Thermo Fisher Scientific). To biotinylate recombinant proteins, the BirA expression vector was cotransfected, and biotin was supplemented at 100 μM in the culture medium. Supernatants from transfected cells were harvested on day 5 after transfection, and recombinant proteins were purified using Ni–nitrilotriacetic acid agarose (QIAGEN). To generate fluorochrome S protein probes, Avi-tag–biotinylated recombinant RBD and spike proteins were conjugated with subsequent fluorochrome-labeled streptavidin at a 4:1.5 ratio overnight at 4°C as follows: spike with streptavidin-Allophycocyanin (APC) (Thermo Fisher Scientific), streptavidin–phycoerythrin (PE) (Thermo Fisher Scientific), or TotalSeq-C0951 PE streptavidin (BioLegend), RBD with streptavidin-APC (Thermo Fisher Scientific), streptavidin-PerCP-Cy5.5 (BioLegend), or TotalSeq-C0953 PE streptavidin (BioLegend).

### Flow cytometry

PBMCs were stained with spike/RBD probes in DMEM supplemented with 2% FBS and 10 μM biotin for 30 min at room temperature. This was followed by staining with the following anti-human antibodies: TotalSeq-C0251 Hashtag 1 (LNH-94, BioLegend), TotalSeq-C0252 Hashtag 2 (LNH-94, BioLegend), TotalSeq-C0253 Hashtag 3 (LNH-94, BioLegend), TotalSeq-C0254 Hashtag 4 (LNH-94, BioLegend), TotalSeq-C0255 Hashtag 5 (LNH-94, BioLegend), TotalSeq-C0256 Hashtag 6 (LNH-94, BioLegend), TotalSeq-C0257 Hashtag 7 (LNH-94, BioLegend), TotalSeq-C0258 Hashtag 8 (LNH-94, BioLegend), TotalSeq-C0181 CD21 (Bu32, BioLegend), TotalSeq-C0154 CD27 (O323, BioLegend), TotalSeq-C0829 CD307e (FcRL5) (509f6, BioLegend), IgA–fluorescein isothiocyanate [polyclonal rabbit F(ab′)2, Dako], IgG-BV421 (G18-145, BD Biosciences), CD2-BV510 (RPA-2.10, BioLegend), CD4-BV510 (RPA-T4, BioLegend), CD10-BV510 (HI10a, BioLegend), CD14-BV510 (M5E2, BioLegend), CD27-BV605 (O323, BD Biosciences), FcRL5-BV650 (509F6, BD Biosciences), CD19-BUV395 (HIB19, BD Biosciences), CD19-BV421 (HIB19, BD Biosciences), CD20-BUV496 (2H7, BD Biosciences), IgM-BUV563 (UCH-B1, BD Biosciences), IgD-PECy7 (IA6-2, BD Biosciences), CD11c-BUV615 (3.9, BD Biosciences), CD21-BUV737 (B-ly4, BD Biosciences), CD62L-BUV805 (SK11, BD Biosciences), and the LIVE/DEAD Fixable Yellow Dead Cell Stain kit (Thermo Fisher Scientific), and 7-Amino-Actinomycin D (7-AAD) (BioLegend). The stained cells were analyzed and sorted using a FACSAria III or a FACS Symphony S6 (BD Biosciences). Data were analyzed using FlowJo software (BD Biosciences).

### Single B cell culture

RBD-binding B_mem_ cells were sorted into 96-well plates at one cell per well using a FACS Symphony S6 (BD Biosciences) and cocultured with feeder cells (MS40L-low) (*39*) in RPMI 1640 medium supplemented with 10% FBS, 55 μM 2-mercaptoethanol, penicillin (100 U/ml), streptomycin (100 μg/ml), 10 mM Hepes, 1 mM sodium pyruvate, 1% minimum essential medium Non-essential Amino Acids Solution (NEAA), recombinant human interleukin-2 (IL-2) (50 ng/ml; Peprotech), recombinant human IL-4 (10 ng/ml; Peprotech), recombinant human IL-21 (10 ng/ml; Peprotech), and recombinant human B-cell activating factor (BAFF) (10 ng/ml; Peprotech). The plates were incubated at 37°C in a humid atmosphere with 5% CO_2_. The medium was half replenished on days 4, 8, 12, 15, and 21, and the supernatants were harvested on day 24.

### mAb generation

Recombinant mAbs were prepared as previously described (*40*, *41*). Briefly, the V_H_/V_L_ genes of spike- and RBD-binding B cell clones identified by LIBRA-seq analysis were commercially synthesized and cloned into expression vectors with human IgG1 heavy chain and kappa/lambda light chain. Pairs of heavy- and light-chain vectors were transfected into Expi293F cells according to the manufacturer’s instructions. Thereafter, the antibodies were purified from the culture supernatant using a protein G column (Thermo Fisher Scientific) and subjected to further analysis after dialysis with PBS.

### Enzyme-linked immunosorbent assay

F96 Maxisorp Nunc-Immuno plates (Thermo Fisher Scientific) were coated with 2 μg/ml of either original RBDs or RBD mutants overnight at 4°C. After washing with PBS, the plates were blocked with 1% bovine serum albumin (BSA) in PBS for 1.5 hours at room temperature. Heat-inactivated plasma and mAbs were serially diluted in PBS containing 1% BSA and 0.05% Tween 20 [eight fourfold serial dilutions starting at 1:20 dilution for plasma; 1:10, 1:20, 1:80, and 1:320 dilutions for prepandemic plasma; or eight fourfold serial dilutions starting at a concentration (1 μg/ml) for mAbs] and then incubated for overnight at 4°C. The following day, the plates were washed with PBS containing 0.05% Tween 20. To determine the avidity index of RBD IgGs, the plates were treated with 7 M urea for 30 min at room temperature before the addition of horseradish peroxidase (HRP)–conjugated goat anti-human IgG (Southern Biotech). HRP activity was visualized with Can Get Signal #2 (TOYOBO), followed by the addition of o-phenylenediamine dihydrochloride (OPD) substrate (Sigma-Aldrich), and optical density at 490 nm (OD490) was measured using an iMark microplate reader (Bio-Rad) and Epoch2 (BioTek). To quantify IgG titers in plasma samples, COVA1-18 or CR3022 was used as a reference antibody. The IgG slopes between two time points were calculated by dividing the difference in IgG titers by the time difference.

### Pseudo-virus production

A VSV pseudo-virus bearing the SARS-CoV-2 spike protein was generated as described previously (*42*, *43*). Briefly, cDNAs of the SARS-CoV-2 spike proteins derived from the Wuhan strain and Beta variant TY8-612 were synthesized (Integrated DNA Technologies Inc.) and cloned into the pCAGGS expression vector. A plasmid (pCAG-SARS-CoV-2) comprising 19 amino acid truncations at the C terminus of the spike protein was constructed. The pCAG-SARS-CoV-2 expression vector was transfected into 293 T cells on collagen-coated tissue culture plates. After 24 hours of incubation, the cells were infected with G-complemented VSVΔG/Luc at a multiplicity of infection of 0.5, and, thereafter, the uninfected viruses were washed. After 24 hours of incubation, the culture supernatants with VSV pseudo-virus were collected, centrifuged to remove cell debris, and then stored at −80°C until use in the virus neutralization assay.

### Virus neutralization assay

For the pseudo-virus neutralization assay, SARS-CoV-2 pseudo-virus was incubated with an equal volume of serially diluted culture supernatants and mAbs (10-fold serial dilutions starting at a 1:4 dilution for culture supernatants and at 5 μg/ml for mAbs) for 1 hour at 37°C. The mixture was inoculated with VeroE6/TMPRSS2 cells seeded in 96-well white flat-bottom plates (Corning and Sumitomo Bakelite) and then incubated for 24 hours at 37°C in a chamber supplied with 5% CO_2_. Luciferase activity in the cultured cells was measured using the Bright-Glo Luciferase Assay Ssystem (Promega) with a GroMax Navigator Microplate Luminometer (Promega). The IC_50_ were calculated using Prism 9 (GraphPad). NT activities in the culture supernatants were determined as positive when the luciferase signals reduced below 50% of the control. For the authentic virus neutralization assay, SARS-CoV-2 virus (Wuhan; 100 median tissure culture infectious dose/ml) was incubated with an equal volume of serially diluted heat-inactivated plasma (threefold serial dilutions starting at 1:2.5 or 1:10 dilutions) for 4 hours at 37°C. The mixture was inoculated with VeroE6/TMPRSS2 cells seeded in 96-well flat-bottom plates (Corning) and then incubated for 60 hours at 37°C in a chamber supplied with 5% CO_2_. The frequencies of live cells were determined using a Cell Counting Kit-8 (Sigma-Aldrich) with an iMark microplate reader (Bio-Rad). The IC_50_ were calculated using Prism 9 (GraphPad)

### Electrochemiluminescence immunoassay

IgG titers in B cell culture supernatants to mutant RBDs were measured using the U-PLEX kit (Meso Scale Discovery) as described previously (*30*). Briefly, biotinylated RBDs were conjugated to linker proteins from the kit by incubation at room temperature for 30 min, followed by another 30-min incubation with stop solution. All the linker-conjugated RBDs were mixed, transferred into U-PLEX plates, and incubated at 4°C overnight. The plates were washed with washing buffer (PBS supplemented with 0.05% Tween 20) three times and filled with MSD Blocker A reagent, followed by 1 hour of incubation at room temperature with rotation for blocking. The plates were washed with the washing buffer three times and were incubated with samples diluted in Diluent 100 (Meso Scale Discovery) at room temperature for 2 hours with rotation. The plates were washed three times and incubated with SULFO-TAG–conjugated anti-human IgG (Meso Scale Discovery) at room temperature for 1 hour with rotation. The plates were washed with the washing buffer and filed with MSD Gold Read Buffer B (Meso Scale Discovery), and electrochemiluminescence was immediately measured with MESO QuickPlex SQ120 (Meso Scale Discovery). Signals were normalized to those of CR3022-IgG1, and a threshold of susceptibility to the mutated RBDs was set as a <0.25 ratio to Wuhan RBD.

### 10x Genomics single-cell processing and next-generation sequencing

10x Genomics single-cell immune profiling technology was used for single-cell sequencing of antigen-specific B cells. Single-cell suspensions were loaded onto the GEM channels of the Chromium Controller microfluidics device (10x Genomics) and processed following the manufacturer’s protocol for target cell recovery of at most 10,000 B cells. Single-cell 5′ RNA sequencing (RNA-seq) and BCR sequencing (BCR-seq) libraries were prepared using the Chromium Next GEM Single Cell 5′ Kit v2 (10x Genomics) and the Chromium Single Cell Human BCR Amplification Kit (10x Genomics), respectively. The antigen, Cellular Indexing of Transcriptomes and Epitopes by Sequencing (CITE-seq), and hashtag antibody libraries were prepared and amplified using a 5′ Feature Barcode Kit (10x Genomics). All library preparations were performed according to the manufacturer’s protocols, and the final libraries were assessed using a LabChip GX Touch (PerkinElmer) instrument to verify the cDNA fragment size. The 5′ RNA-seq, BCR-seq, and feature barcode libraries were analyzed, quantified, and sequenced using DNBSEQ-G400 (MGI). This resulted in approximately 50,000 reads per cell for each 5′ RNA-seq library.

### Cell filtering, demultiplexing, and UMAP

The raw reads were processed using Cell Ranger 4.0 (10x Genomics). Gene expression–based clustering was performed using the Seurat R package (v4.0) (*44*). Briefly, cells with a mitochondrial content of >10% and cells with <750 or >3500 detected genes were considered outliers (dying cells and empty droplets and doublets, respectively) and filtered out. Hashtag oligo demultiplexing was performed on centered log ratio–normalized hashtag unique molecular identifier counts, and clonotypes were matched to the gene expression data through their droplet barcodes using Python scripts. Only cells assigned a single hashtag, and a BCR heavy chain clonotype was retained for downstream analyses. The Seurat SCTransform function was used for normalization, and the data from the three experimental batches were integrated without performing batch-effect correction. Last, UMAP two-dimensional embeddings were computed using the first 50 principal components analysis components and the RunUMAP function in Seurat (parameters: dims = 1.15, n.neighbors = 8, min.dist = 0.05).

### CITE-seq normalization

Surface marker antibody counts were normalized in Seurat using the centered log ratio transformation and then scaled and centered using Seurat’s ScaleData function for each dataset before merging.

### LIBRA normalization, scaling, and dim/bright definition

The probe counts were log_10_-transformed, standardized, and scaled to unit variance using the StandardScaler function of the scikit-learn Python library. Healthy control (no probe staining) cells were used to define a probe-positive/negative threshold, and cells with lower intensity, below 33 percentiles in the positive compartment, were further defined as dim. The remaining cells with higher intensity were defined as bright.

### SELL high/low definition

*SELL* gene expression was calculated on a per-cell basis by normalizing the *SELL* Unique molecular identifier (UMI) counts to the total UMI counts, multiplying by a scale factor (10,000), and performing log transformation using the NormalizeData function of Seurat with its default parameters. A high/low threshold of 2 was arbitrarily chosen, as it resulted in a low/high *SELL* ratio close to 50:50 within cells where *SELL* was detected (1 or more UMI).

### Clonotype/clonal assignment and mutation count calculation

Single-cell clonotype assignment was initially performed using Cell Ranger (10x Genomics). The consensus sequence of each heavy-chain clonotype was then exported and used for clonal assignment. Clones were defined per donor, and their germline sequence was reconstructed using the DefineClones and CreateGermlines functions of the Change-O package of the Immcantation suite (*45*). The number of somatic hypermutations was then calculated by comparing each clonotype sequence with its corresponding germline sequence, both at the DNA and amino acid levels. Last, for some selected clones of interest, lineage tree reconstruction and plotting were performed using the Alakazam package of the Immcantation suite.

### Clonal lineage

Unmutated common ancestors and phylogenic relationships for clonal lineages were inferred using CloAnalyst software (*46*). Phylogenetic trees were visualized using the R software package.

### Ethical statement approval

All studies were approved by the institutional review board of the National Institute of Infectious Diseases (#913, #1218, #1299, and #1321). This study was conducted in accordance with the principles of the Declaration of Helsinki. All volunteers provided written informed consent before enrollment.

### Statistical analysis

Data were analyzed using GraphPad Prism 9 (GraphPad Software Inc., CA, USA). Two-tailed Mann-Whitney *U* tests, two-tailed Wilcoxon matched-pairs signed-rank tests, two-sided Kruskal-Wallis test with subsequent Dunn’s multiple comparisons test, Fisher’s exact test, chi-square test, and Spearman’s rank-order correlation test were used for statistical analysis as indicated in the figure legends. Unless otherwise indicated, the data in the figures are presented as geometric means. Jackknife resampling analysis was done by bootstrap package boot_1.3-28 in R v 4.2.2.

## Notes

View/request a protocol for this paper from *Bio-protocol*.
