# Supplementary material for: CD62L expression marks SARS-CoV-2 memory B cell subset with preference for neutralizing epitopes
Source: Sci Adv. 2023 Jun 14;9(24):eadf0661. doi: 10.1126/sciadv.adf0661 (PMC10266721; doi:10.1126/sciadv.adf0661)
Supplement: Supplementary file 1 — Figs. S1 to S5 [file sciadv.adf0661_sm.pdf]

Science Advances
AAAS

Supplementary Materials for

CD62L expression marks SARS-CoV-2 memory B-cell subset with preference for neutralizing epitopes

Taishi Onodera *et al.*

Corresponding author: Yoshimasa Takahashi, ytakahas@niid.go.jp; Kazuo Yamashita, yamashita@kotai-bio.com

Sci. Adv. **9**, eadf0661 (2023)
DOI: 10.1126/sciadv.adf0661

This PDF file includes:

Figs. S1 to S5

Supplementary Materials

Figs. S1 to S5

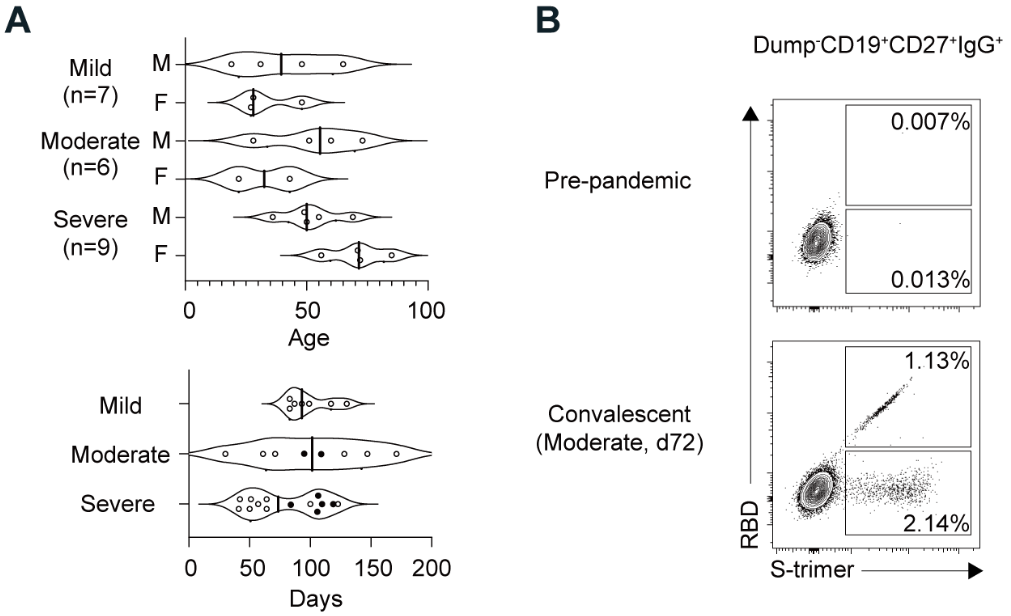

Fig. S1. The participant demographics and flow cytometric gating for S-trimer/RBD-binding B cells

(A) The participant demographics and days after the disease onset are summarized. The days of second blood collections were indicated by closed circles. **(B)** S-trimer/RBD probes were applied to COVID-19-convalescent samples and pre-pandemic samples for validating the staining specificity.

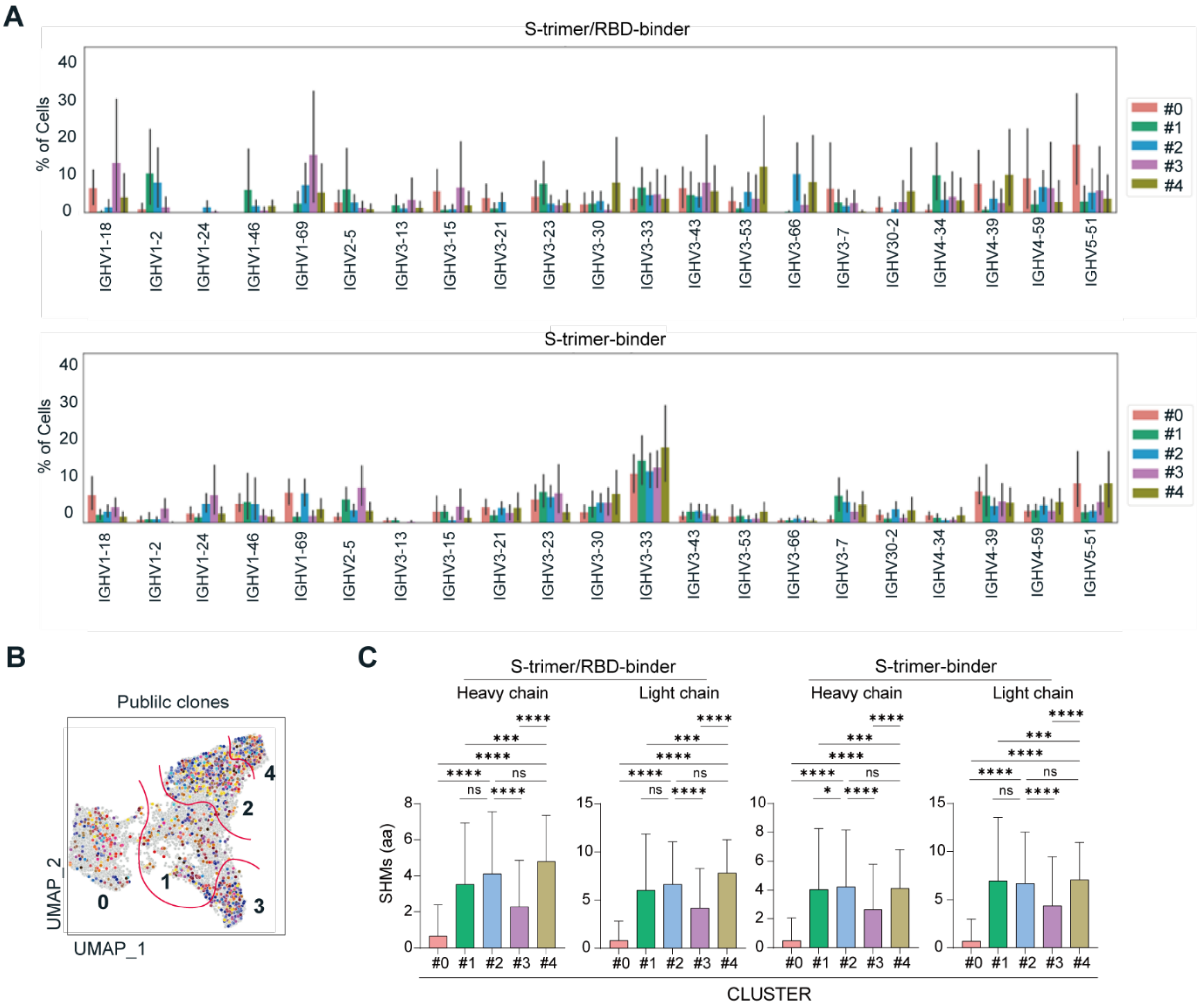

Fig. S2. Cluster distribution of V_H gene repertoire, public clones, and numbers of somatic hypermutations from RBD binders and non-RBD binders among S-trimer binders

(A) Heavy chain V-gene fraction for each cluster split by specificities. Upper and lower panels show RBD binders and non-RBD binders, respectively. **(B)** Distribution of public clusters projected onto the UMAP. Different colors indicate different donors. **(C)** Number of somatic hypermutations (SHMs) against amino acid sequences of germline V-genes for each chain. The SHMs were counted for each cluster and specificity. Statistical analyses were performed using the Kruskal-Wallis test followed by Dunn's multiple comparison test in (C) [*$P < 0.05$, ***$P < 0.001$, ****$P < 0.0001$, ns (not significant; $P \geq 0.05$)].

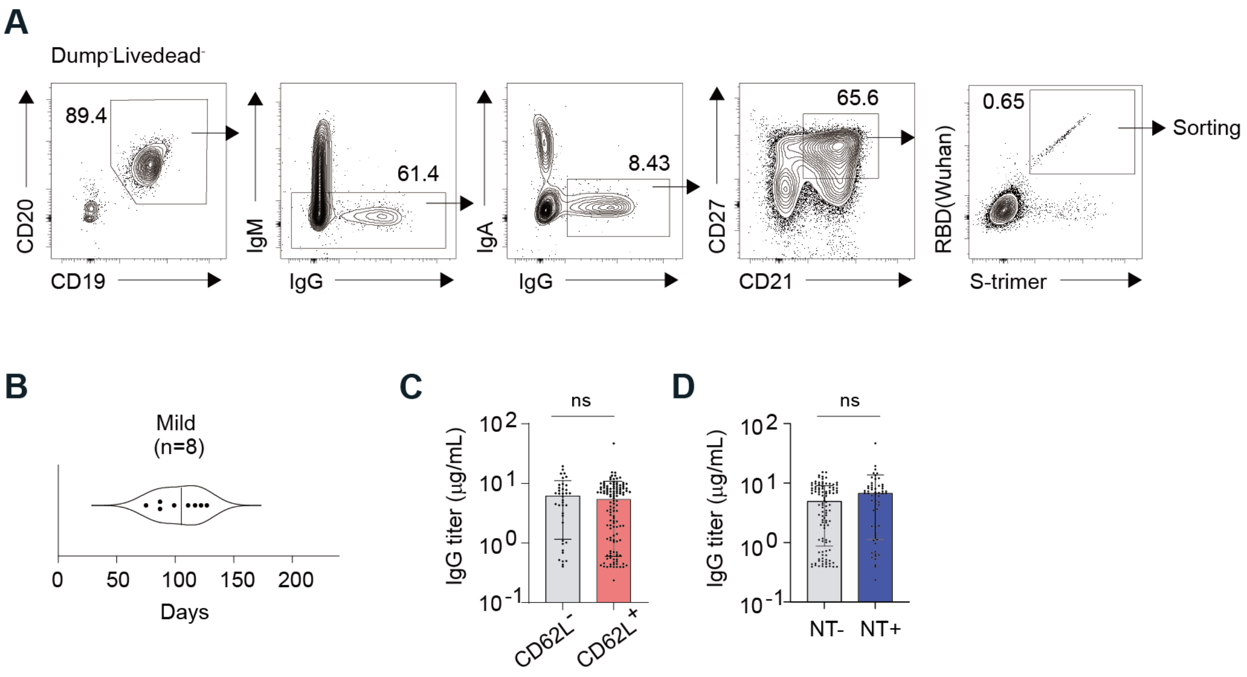

Fig. S3. Flow cytometric gating, participant demographics, and IgG production from single-cell culture analysis

(A) FACS gating strategy for single-cell culture sorting is presented. **(B)** The participants demographics and days after the disease onset are summarized (*n*=8). **(C)** IgG concentrations in culture supernatants from the indicated B_{mem} cell subsets are plotted. **(D)** IgG concentrations with or without NT activities are plotted. Statistical analyses were performed using the Mann-Whitney test in (C) and (D) (ns, not significant; $P \geq 0.05$).

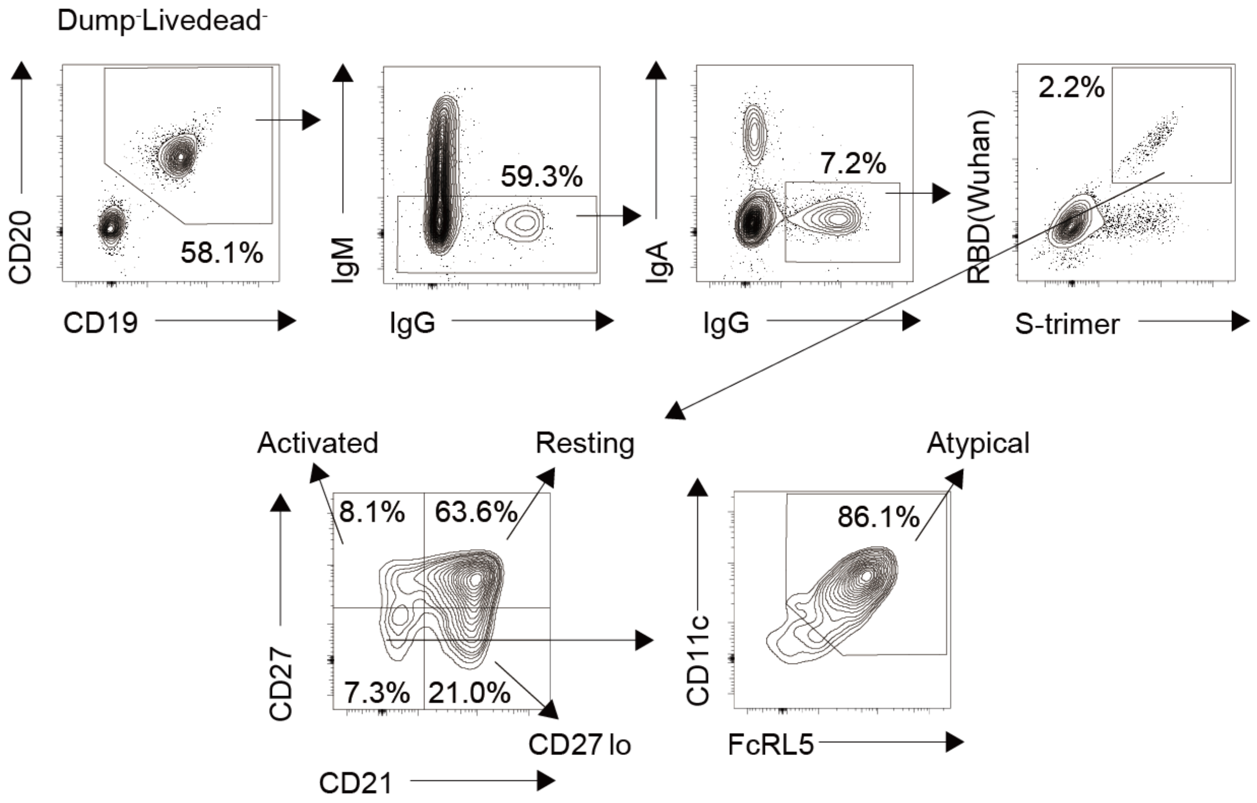

Fig. S4. The gating strategies for four Bmem subsets among IgG$^+$RBD-binders.

RBD-binding IgG$^+$ B$_{mem}$ cells were subdivided into CD27$^+$CD21$^+$ (resting), CD27^{low}/CD21$^+$ (CD27^{lo}), CD27$^+$CD21^{low} (activated), and CD27^{low}CD21^{low}CD11c$^+$FcRL5$^+$ (atypical)

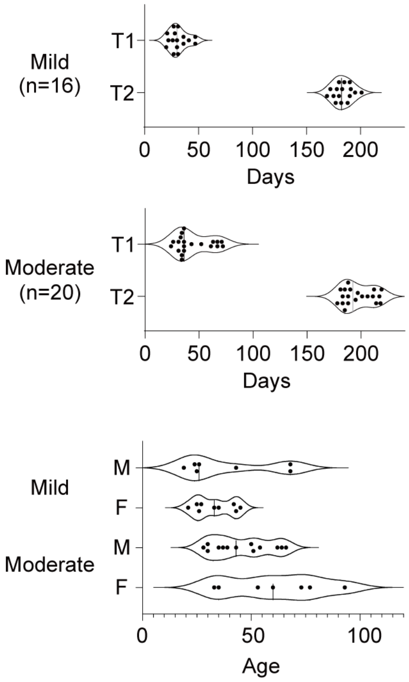

Fig. S5. The participant demographics of COVID-19-convalescent individuals for longitudinal analysis

The days after the disease onset and age of the participants are summarized.
